# Prognostic significance of tumor-associated macrophages polarization markers in lung cancer: a pooled analysis of 5105 patients

**DOI:** 10.1042/BSR20221659

**Published:** 2023-02-02

**Authors:** Bin Yi, Yuanda Cheng, Ruimin Chang, Wolong Zhou, Huili Tang, Yang Gao, Chunfang Zhang

**Affiliations:** 1 Department of Thoracic Surgery, Xiangya Hospital, Central South University, Changsha, 410008 Hunan, P. R. China; 2 Xiangya Lung Cancer Center, Xiangya Hospital, Central South University, Changsha, 410008 Hunan, P. R. China; 3 National Clinical Research Center for Geriatric Disorders, Changsha, 410008 Hunan, P. R. China

**Keywords:** lung cancer, pooled analysis, prognosis, Tumor-associated macrophages

## Abstract

**Background:** The prognostic significance of tumor-associated macrophages (TAMs) in patients with lung cancer (LCa) remains controversial. We therefore conducted the present study to systematically evaluate the role of different TAMs markers and histologic locations on the prognosis of LCa.

**Methods:** Searches of Web of Science, PubMed, and EMBASE databases were performed up to 28 February 2022. The pooled analysis was conducted in random-effect or fixed-effects model with hazard risk (HR) and 95% confidence interval (CI) for survival data including overall survival (OS), and disease-free survival (DFS) from raw or adjusted measures, according to different TAMs markers and histologic locations.

**Results:** Including a total of 5105 patients from 30 eligible studies, the results indicated that the total count of CD68+ TAMs was negatively associated with OS and DFS, which was also observed in the relationship of CD68+ or CD204+ TAMs in tumor stroma (TS) with OS and DFS (all *P*<0.05). Conversely, higher CD68+ TAMs density in tumor nest (TN) or TN/TS ratio of CD68+ TAMs predicted better OS (all *P*<0.05). Similarly, higher HLA-DR+ TAMs density was correlated with better OS in TN and TS (all *P*<0.05). Besides, neither nest CD163+ TAM density nor stromal CD163+ TAM density was a prognostic factor in LCa patients (all *P*>0.05).

**Conclusion:** Our study indicated that different TAMs markers and histologic locations could bring about different prognostic effects in LCa patients. Great understanding of the infiltration modes of TAMs may contribute to improve outcomes of LCa patients.

## Introduction

Lung cancer (LCa), one of the most common cancer among malignant diseases, is the leading cause of cancer death in the world [1]. With the growth of geriatric population, LCa, a risk to human health, further aggravates global disease burden [2]. Although encouraging advances have been made in the diagnosis and treatment of LCa, the overall survival (OS) is still not optimistic, especially for patients with advanced tumors [3]. Some established prognostic indicators, including TNM classification scheme, histological grade, and epidermal growth-factor receptor (EGFR), remain not unsatisfactory for revealing the biological characteristics and prognosis of LCa [4–6]. Therefore, new biomarkers are essential to investigate for reflecting tumor progression and prognosis in LCa patients.

Recently, it is reported that tumor microenvironment (TME), which is regarded as a prognostic biomarker, plays an important part in LCa progression, invasion, and metastasis [7]. Tumor-associated macrophages (TAMs) are the main component in TME, accounting for approximately 50% of TME cells [8]. Moreover, TAMs are identified as two main functional subtypes based on their immune responses, of which M1 TAMs could activate antitumor immunity and exert cytotoxic effects on cancer cells, and M2 TAMs could promote tumor cell growth, invasion, and metastasis [9]. Several studies have demonstrated the prognostic value of TAMs in various cancers, including lung [10,11], breast [12], and gastric cancer [13]. In general, high infiltration of TAMs indicates a poor prognosis; however, the conclusions vary across different subsets and distribution of TAMs. Therefore, the aim of the present study was to perform a pooled analysis to evaluate the effect of different TAMs markers and histologic locations on the prognosis of LCa.

## Methods

### Search strategy

The present study was registered with PROSPERO (CRD42022323957). The present study was performed in accordance with the Preferred Reporting Items for Systematic Reviews and Meta‐Analyses (PRISMA) guidelines [14]. Two investigators (W.Z. and H.T.) independently searched the Web of Science, PubMed, and EMBASE databases for potential studies published in journals until 28 February 2022. The following Mesh terms were used: ‘macrophage,’ ‘tumor-associated macrophage,’ ‘TAM,’ ‘pulmonary,’ and ‘lung.’ We also undertaken forward and backward citation tracking for avoiding miss any possible literature. No language or country limitations were applied to the present pooled analysis. All studies reporting TAMs and LCa were included and screened by two authors independently based on the inclusion criteria.

### Inclusion criteria

We included the study reporting TAMs associated with LCa. Studies were eligible for inclusion met all of the following criteria: (1) patients with LCa were diagnosed by pathology; (2) patients included in the study should not diagnosed with any previous cancer history; (3) TAMs had to be measured at the primary tumor site using immunohistochemistry (IHC) with the markers, such as CD68, HLA-DR, CD163, and CD204; (4) the study design was a cohort study, either prospective, retrospective, or case control studies, evaluating the association of TAMs with OS or disease-free survival (DFS).

### Exclusion criteria

We excluded the study measuring TAMs at metastases or local relapse site. In addition, a study in specific types of literature, such as reviews, comments, and conference abstracts, was also excluded from our study.

### Data extraction and quality assessment

Two reviewers independently extracted relevant data from the original studies using standardized data extraction form and clarified discrepancies by re-evaluation and discussion with the other authors. We extracted the following data for analysis: name of the first author, publication year, country, demographic characteristics of patients, study period, macrophage markers, macrophage distribution [tumor nest (TN) or tumor stroma (TS)], tumor type, tumor stage, OS, and DFS with adjusted or unadjusted hazard ratios (HRs) and 95% confidence interval (CI). TAMs in the TN were defined as intraepithelial tumor-infiltrating macrophages, and TS was defined as the stromal tissue surrounding the tumor nest. We also collected the prognostic information from study only reported with a Kaplan–Meier (KM) plot and a *P*-value derived from log-rank analysis. HRs and 95% CI were extracted from KM plot using Engauge Digitizer version 4.1 (free software downloaded from http://sourceforge.net) and calculated as previously described [15]. We used the low macrophage-infiltrating group as a reference to calculate HR. If the high macrophage-infiltrating group was used as reference in the article, then the association measure and CI were inverted. The corresponding author of study was contacted to request any unclear or missing data.

Two experienced researchers independently assessed the quality for each included study using the modified Newcastle-Ottawa Scale (NOS) based on the current PRISMA guidelines [16]. The researchers focused on measurement and selection bias because most studies included in the present review were cross-sectionally designed. Studies obtained a score based on three evaluation indicator including patient selection, study comparability, and outcome assessment. The included study was graded as high quality with an NOS score ≥ 6. Disagreements were resolved by a third person who served as an intermediary and made the final decision.

### Statistical analysis

The statistical analysis was performed according to the recommendations from the Cochrane Collaboration. The HRs with 95% CI were used to evaluate the correlation between the TAMs density and survival. Heterogeneity across studies was assessed by the *I^2^* statistic. If *I^2^*≥50%, which indicates significant differences, a random effects model was utilized. Conversely, a fixed effects model was used if *I^2^*<50%, which indicates no significant differences.

Sensitivity analysis was conducted through observing the impact of changing the statistical method and analysis model. When the number of articles available was >5, potential publication bias was assessed by the symmetry of funnel plot. Review Manager Version 5.3 (The Nordic Cochrane Center, The Cochrane Collaboration, 2014, Copenhagen) software was used to analyze the pooled data. A two-tailed *P*-value <0.05 was considered statistically significant.

## Results

### Search results

**Figure 1:**
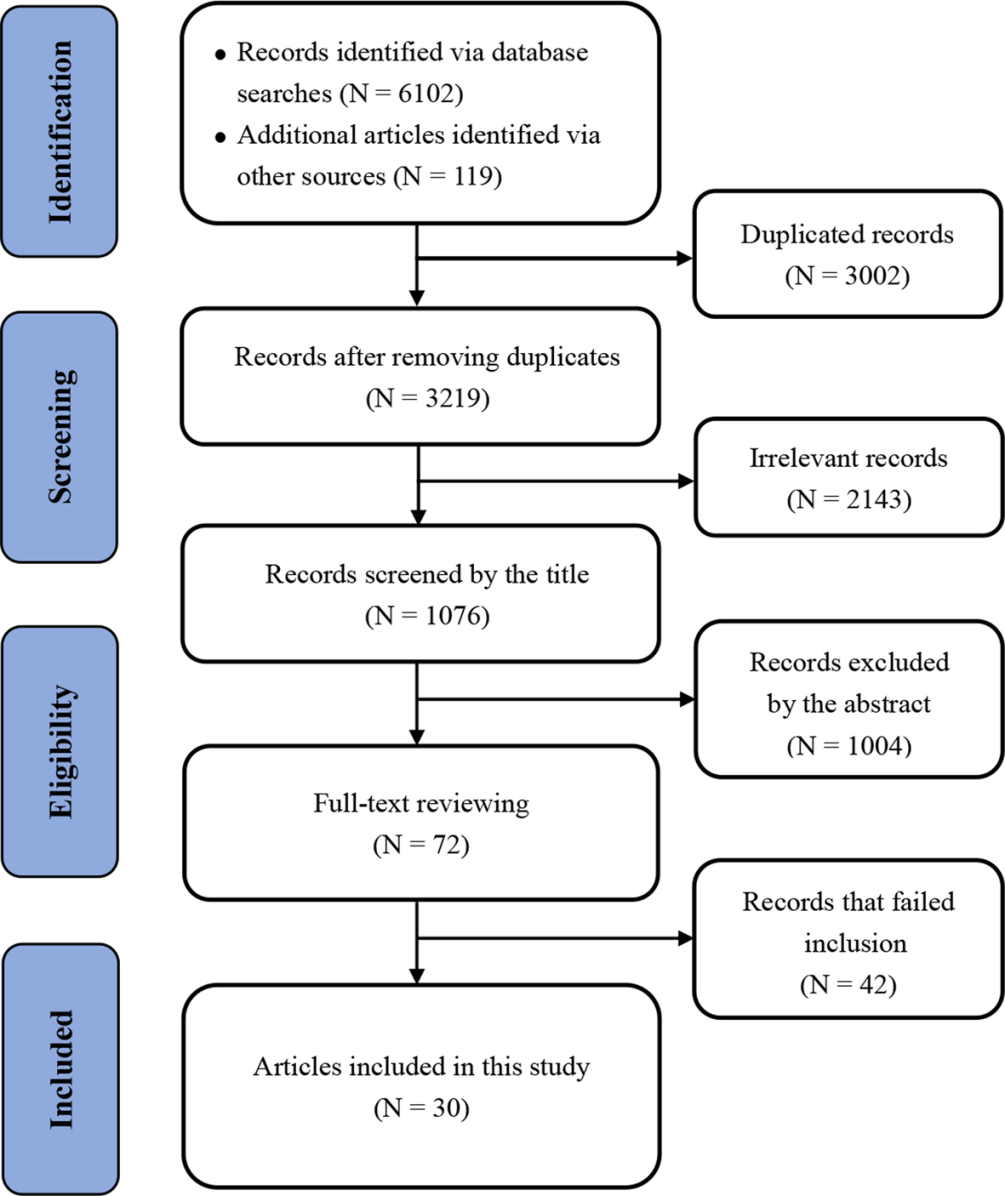
Flow diagram of the studies’ selection

A total of 6221 articles were found during our initial search. After electronically removing 5145 duplicated articles and irrelevant studies, 1004 studies were excluded by reading the title and abstract, and 72 articles were evaluated in detail. Then, 42 articles were excluded after reviewing the full text, 30 unique articles were ultimately included in this pooled analysis [10,11,17–44]. The study searching and inclusion procedure is presented in Figure 1.

### Basic characteristics and quality assessment

The main characteristics of the included studies are summarized in Table 1. We included 30 studies in our pooled analysis [10,11,17–44], which published between 1999 and 2021 and conducted in ten countries from 1978 to 2018 (Finland, Japan, China, UK, Republic of Korea, Norway, Brazil, Denmark, France, Germany). A total of 5105 patients were included, with the reported age from 19 to 91 years across eligible studies.

**Table 1:** Characteristics of studies included in the pooled analysis

Author (published year)	Country	Study period	Sample size	Male	Age	Markers	Tissue distribution	Type	Stage	Outcome assessment	NOS
Eerola (1999) [17]	Finland	1978–1995	38	35	39–78 y	CD68	Tumor nest	LCLC	I–III	OS	7
Takanami (1999) [18]	Japan	1986–1992	113	66	Mean 62 y (30–79 y)	CD68	Unavailable	ADC	I–IV	OS	7
Chen (2003) [19]	Taiwan	1994.05–1994.12	35	24	Mean 60.3 y	CD68	Unavailable	ADC,SCC	I–IIIA	OS	7
Chen (2005) [20]	Taiwan	1994.09–1996.09	41	27	Mean 60 y	CD68	Unavailable	ADC,SCC	I–IV	DFS	7
Welsh (2005) [21]	UK	1991–1994; 1999.01–1999.12	175	116	Mean 67.7 y (39–91 y)	CD68	Tumor nest and stroma	NSCLC	I–IV	OS	9
Ho (2008) [22]	Taiwan	1996.09–1998.09	68	40	Unavailable	TREM-1, CD68	Unavailable	NSCLC	I–III	OS, DFS	7
Kawai (2008) [23]	Japan	1996.01–2004.12	199	139	Mean 62 y (39–79 y)	CD68	Tumor nest and stroma	NSCLC	IV	OS	8
Kim (2008) [24]	Korea	1997.01–1998.12	144	106	Mean 60.4 y	CD68	Tumor nest and stroma	NSCLC	I–IV	OS	9
Al-Shibli (2009) [25]	Norway	1990–2004	335	253	Mean 67 y (28–85 y)	CD68	Tumor stroma	NSCLC	I–III	OS	8
Ohri (2009) [26]	UK	1991–1994; 1999.01–1999.12	40	16	Unavailable	CD68, CD163, HLA-DR	Tumor nest and stroma	NSCLC	I–IV	OS	8
Dai (2010) [27]	China	1999.08–2001.08	99	80	66 y (37–80 y)	CD68	Tumor nest and stroma	NSCLC	I–IV	OS	8
Ma (2010) [28]	China	1999.06–2001.08	100	81	Unavailable	CD68, CD163, HLA-DR	Tumor nest and stroma	NSCLC	I–IV	OS	9
Ohtaki (2010) [29]	Japan	1996.01-1998.03	170	85	Mean 62 y (33–85 y)	CD68, CD204	Tumor stroma	ADC	I–IIIA	OS	8
Zhang (2011) [30]	China	2003–2006	65	38	Mean 51.5 y (32–76 y)	CD68	Tumor nest and stroma	ADC	I–IV	OS	9
Hirayama (2012) [31]	Japan	2000.01–2006.12	208	188	Unavailable	CD204	Tumor nest and stroma	SCC	I–IIIA	OS, DFS	8
Souza (2012) [32]	Brazil	Unavailable	65	39	Mean 62 y (34–82 y)	CD68	Unavailable	NSCLC	I–III	OS	6
Carus (2013) [33]	Denmark	2003.01–2006.12	335	194	Unavailable	CD163	Tumor nest and stroma	NSCLC	I–IIIA	OS, DFS	7
Feng (2014) [34]	Taiwan	2005–2008	28	15	Mean 59 y (41–78 y)	CD68	Tumor nest and stroma	NSCLC	I–IIIA	OS, DFS	8
Pei (2014) [35]	China	2003–2008	417	231	Unavailable	CD68	Tumor stroma	NSCLC	I–IIIA	OS, DFS	7
Li (2014) [36]	China	2007.01–2008.06	132	86	Mean 58.5 y (38–74 y)	CD163	Tumor nest and stroma	NSCLC	I–IV	OS	8
Li (2015) [37]	China	2003–2006	159	109	Median 61 y (44–77 y)	CD68	Unavailable	NSCLC	I–III	OS, DFS	8
Mansuet-Lupo (2016) [38]	France	2001.06–2005.06	316	225	Median 61 y (19–84 y)	CD68	Unavailable	ADC	I–IV	OS	8
Li (2018) [39]	Japan	2005–2013	297	184	Unavailable	CD68 CD204	Tumor nest and stroma	NSCLC	I–IV	OS	8
Cao (2019) [40]	China	2012–2014	137	77	Median 59 y (34–75 y)	CD68 CD163	Tumor nest and stroma	NSCLC	I–III	OS, DFS	7
Rakaee (2019) [41]	Norway	1990–2010	553	Unavailable	Unavailable	CD68, CD163, CD204, HLA-DR	Tumor nest and stroma	NSCLC	I–III	OS	8
Thielmann(A) (2019) [42]	Germany	2001.04.18–2001.12.04	53	45	Mean 61.7 y	CD68	Unavailable	SCC	I–IV	OS	7
Thielmann(B) (2019) [42]	Germany	2001.04.18–2001.12.04	49	29	Mean 59.9 y	CD68	Unavailable	ADC	I–IV	OS	7
Chen (2020) [43]	China	2006.06–2012.12	213	184	Unavailable	CD163	Tumor stroma	ADC	I–IV	OS	7
Hang (2020) [44]	China	2008.04–2014.01	92	71	Median 61 y (39–75 y)	CD68	Unavailable	NSCLC	I–III	OS	6
Hwang (2020) [10]	Korea	1993–2004; 2010.01–2012.12	349	241	Mean 65.5 y (35–90 y)	CD68 CD163	Tumor stroma	NSCLC	I–IV	OS	7
Amemiya (2021) [11]	Japan	1998.11–2018.09	80	68	Mean 67 y (43–84 y)	CD204	Unavailable	NSCLC	I–IV	OS, DFS	7

Abbreviations: ADC, adenocarcinoma; DFS, disease-free survival; LCLC, large cell lung cancer; NOS: Newcastle-Ottawa Scale checklist; NSCLC, non-small-cell lung cancer; OS, overall survival; SCC, squamous cell carcinoma.

As for TAMs identification, 25 out of 30 studies used CD68 [10,17–30,32,34,35,37–42,44], three studies used HLA-DR [26,28,41], eight used CD163 [10,26,28,33,40,41,43,44], and five studies used CD204 [11,29,31,39,41] macrophages marker to detect TAMs by IHC. Fourteen articles investigated the role of TAMs in both TN and TS [21,23,24,26–28,30,31,33,34,39–41,44], one studies only detected TAMs in TN [17], and five articles only reported TAMs in TS [10,25,29,35,43]. Moreover, 29 studies provided OS data [10,11,17–19,21–44], and nine studies reported DFS data [11,20,22,31,33–35,37,40]. The NOS scores of these studies were ranged from 6 to 9 (Table 1).

### Prognostic significance of CD68+ TAMs

**Figure 2:**
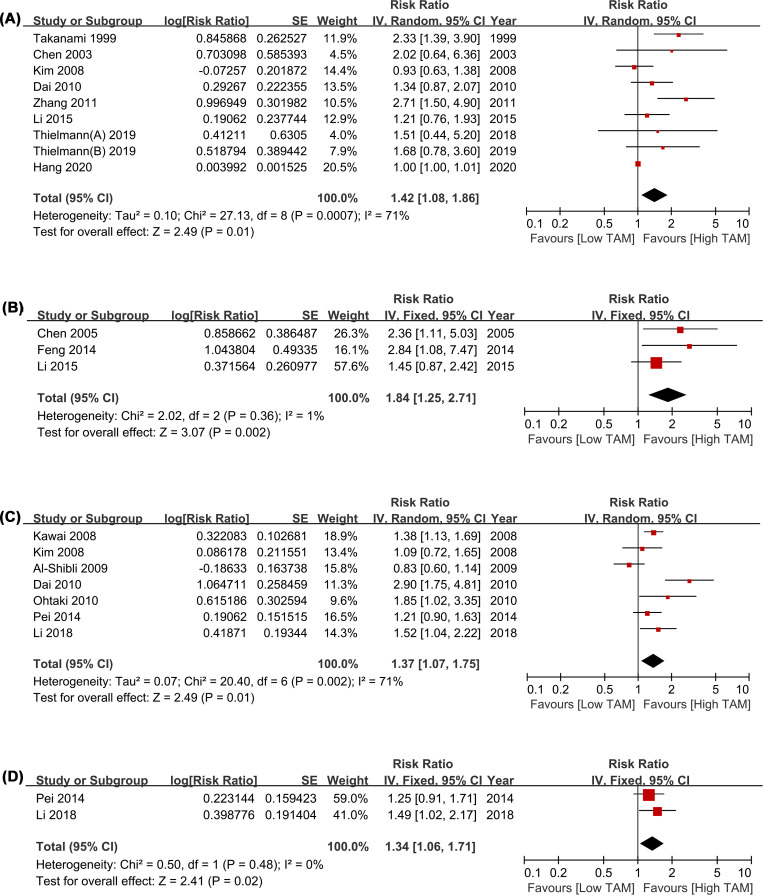
Forest plots comparing the survival of CD68+ TAMs in the tumor and TS for LCa patients (**A**) HR of OS for CD68+ TAMs in the tumor; (**B**) HR of DFS for CD68+ TAMs in the tumor; (**C**) HR of OS for CD68+ TAMs in TS; (**D**) HR of DFS for CD68+ TAMs in TS. Abbreviations: DFS, disease-free survival; HR, hazard risk; LCa, lung cancer; OS, overall survival; TAMs, tumor-associated macrophages; TS, tumor stroma.

A total of 25 studies were included in the analysis of CD68+ TAMs on survival data in patients with LCa [10,17–30,32,34,35,37–42,44]. Compared with low density of total CD68+ TAMs, high density of total CD68+ TAMs was significantly associated with poor OS (HR = 1.42, 95% CI = 1.08–1.86, *P*=0.01; *I^2^* = 71%; Figure 2A) and DFS (HR = 1.84, 95% CI = 1.25–2.71, *P*=0.002; *I^2^* = 1%; Figure 2B). Similarly, high CD68+ TAMs density in TS indicated poor OS (HR = 1.37, 95% CI = 1.07–1.75, *P*=0.01; *I^2^* = 71%; Figure 2C) and DFS (HR = 1.34, 95% CI = 1.06–1.71, *P*=0.02; *I^2^* = 0%; Figure 2D).

**Figure 3:**
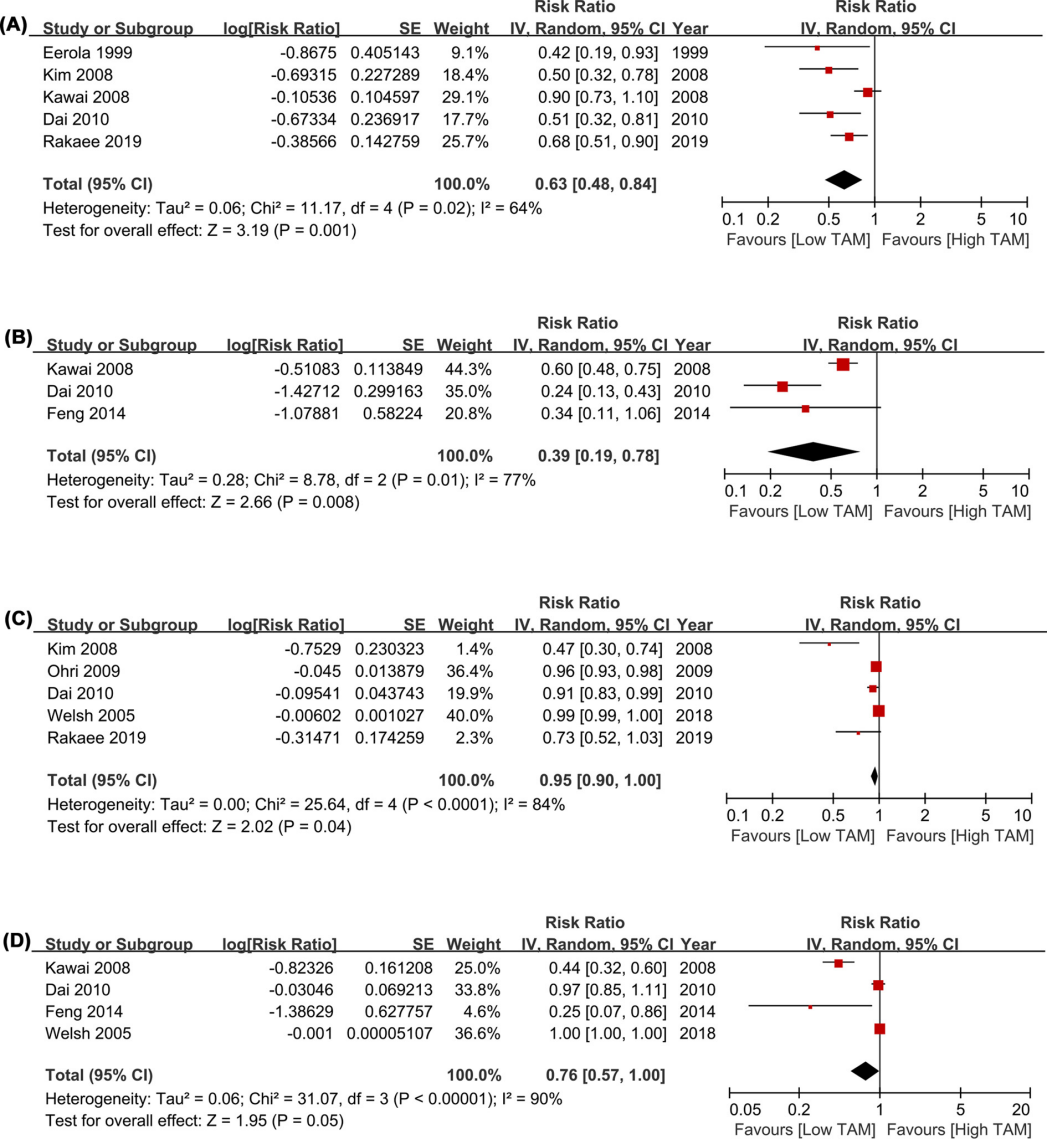
Forest plots comparing the survival of nest and TN/TS CD68+ TAMs for LCa patients (**A**) HR of OS in raw data for CD68+ TAMs in TN; (**B**) HR of OS in raw data for TN/TS CD68+ TAMs; (**C**) HR of OS with adjusted measures for CD68+ TAMs in TN; (**D**) HR of OS with adjusted measures for TN/TS CD68+ TAMs. Abbreviations: HR, hazard risk; LCa, lung cancer; OS, overall survival; TAMs, tumor-associated macrophages; TN, tumor nest; TS, tumor stroma.

However, higher CD68+ TAMs density in TN was significantly associated with better OS (HR = 0.63, 95% CI = 0.48–0.84, *P*=0.001; *I^2^* = 64%; Figure 3A). Moreover, greater TN/TS ratio of CD68+ TAMs predicted better OS (HR = 0.39, 95% CI = 0.19–0.79, *P*=0.008; *I^2^* = 77%; Figure 3B). As for adjusted measurements to OS, the results also supported the significant correlations of better OS with higher CD68+ TAMs density in TN (HR = 0.95, 95% CI = 0.9–1.0, *P*=0.04; *I^2^* = 84%; Figure 3C) and TN/TS ratio of CD68+ TAMs (HR = 0.76, 95% CI = 0.57–1.0, *P*=0.05; *I^2^* = 90%; Figure 3D).

### Prognostic significance of HLA-DR+ TAMs

**Figure 4:**
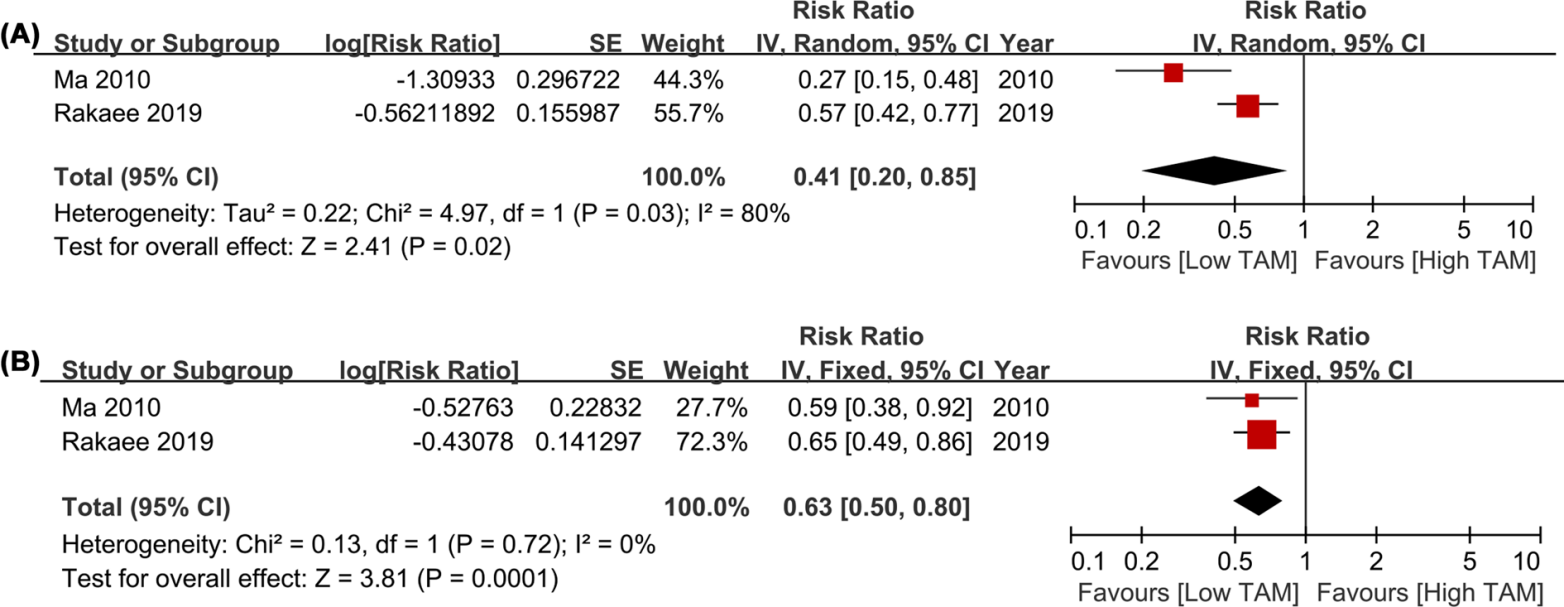
Forest plots comparing the survival of HLA-DR+ TAMs in TN and TS for LCa patients (**A**) HR of OS for HLA-DR+ TAMs in TN; (**B**) HR of OS for HLA-DR+ TAMs in TS. Abbreviations: HR, hazard risk; LCa, lung cancer; OS, overall survival; TAMs, tumor-associated macrophages; TN, tumor nest; TS, tumor stroma.

Given the different heterogeneity, the random-effect model was used in assessing HLA-DR+ TAMs in TN (*I^2^* ≥ 50%), and the fixed effect model was used in assessing HLA-DR+ TAMs in TS (*I^2^ *< 50%). The present pooled analysis indicated that a high HLA-DR+ TAMs density was significantly associated with better OS than a low HLA-DR+ TAMs density in TN with a pooled HR of 0.41 (95% CI = 0.20–0.85, *P*=0.02; *I^2^* = 80%; Figure 4A). In addition, a high HLA-DR+ TAMs density in TS also indicated better OS (HR = 0.63, 95% CI = 0.50–0.80, *P*=0.0001; *I^2^* = 0; Figure 4B).

### Prognostic significance of CD204+ TAMs

**Figure 5:**
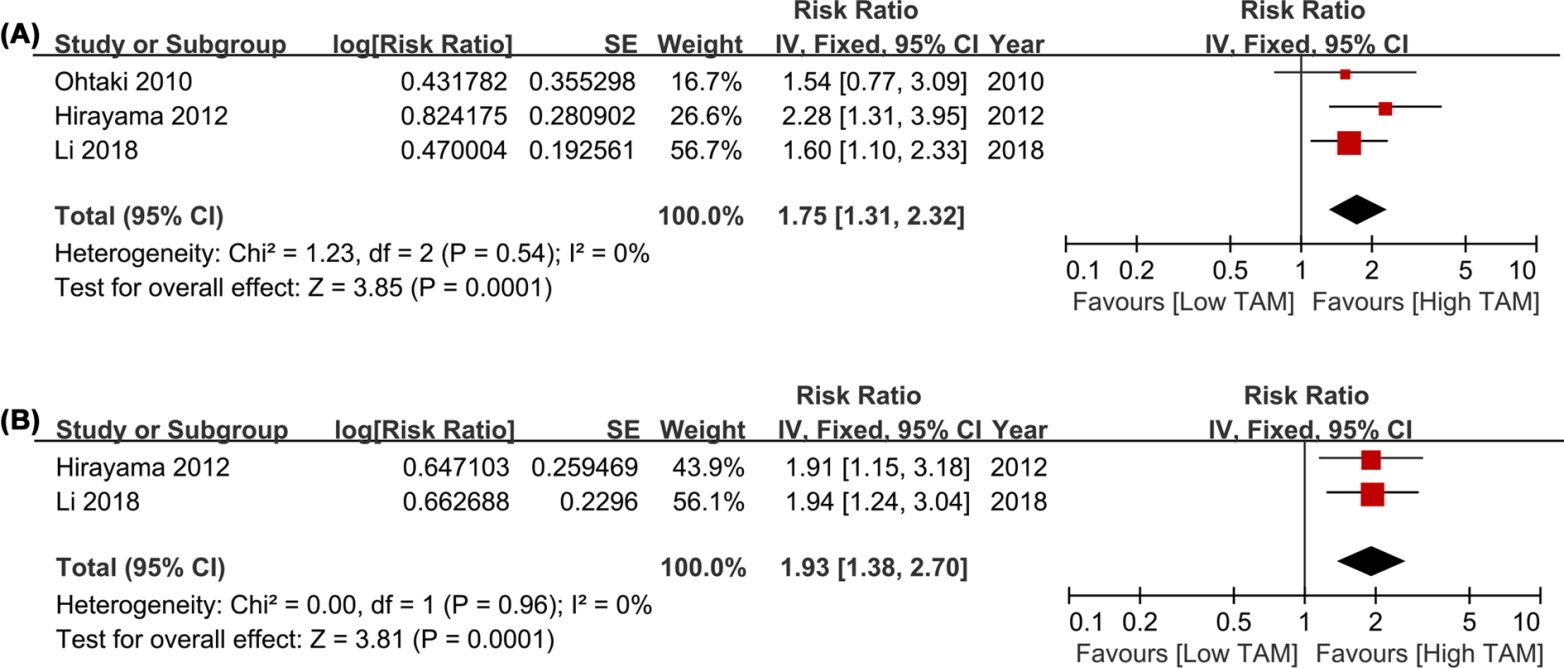
Forest plots comparing the survival of CD204+ TAMs in TS for LCa patients (**A**) HRs of OS in raw data for CD204+ TAMs in TS; (**B**) HRs of OS with adjusted measures for CD204+ TAMs in TS. Abbreviations: HR, hazard risk; LCa, lung cancer; OS, overall survival; TAMs, tumor-associated macrophages; TS, tumor stroma.

This pooled analysis was performed in fixed-effect model for the absent of heterogeneity in the followed results (all *I^2^* < 50%). Relative to low CD204+ TAMs density, high CD204+ TAMs density in TN predicted poor OS in patients with LCa (HR = 1.75, 95% CI = 1.31–2.32, *P=*0.0001; *I^2^* = 0; Figure 5A). Furthermore, the result showed that a high CD204+ TAMs density was significantly associated with poor DFS than a low CD204+ TAMs density in TS with a pooled HR of 1.93 (95% CI = 1.38-2.7, *P=*0.0001; *I^2^* = 0; Figure 5B).

### Prognostic significance of CD163+ TAMs

**Figure 6:**
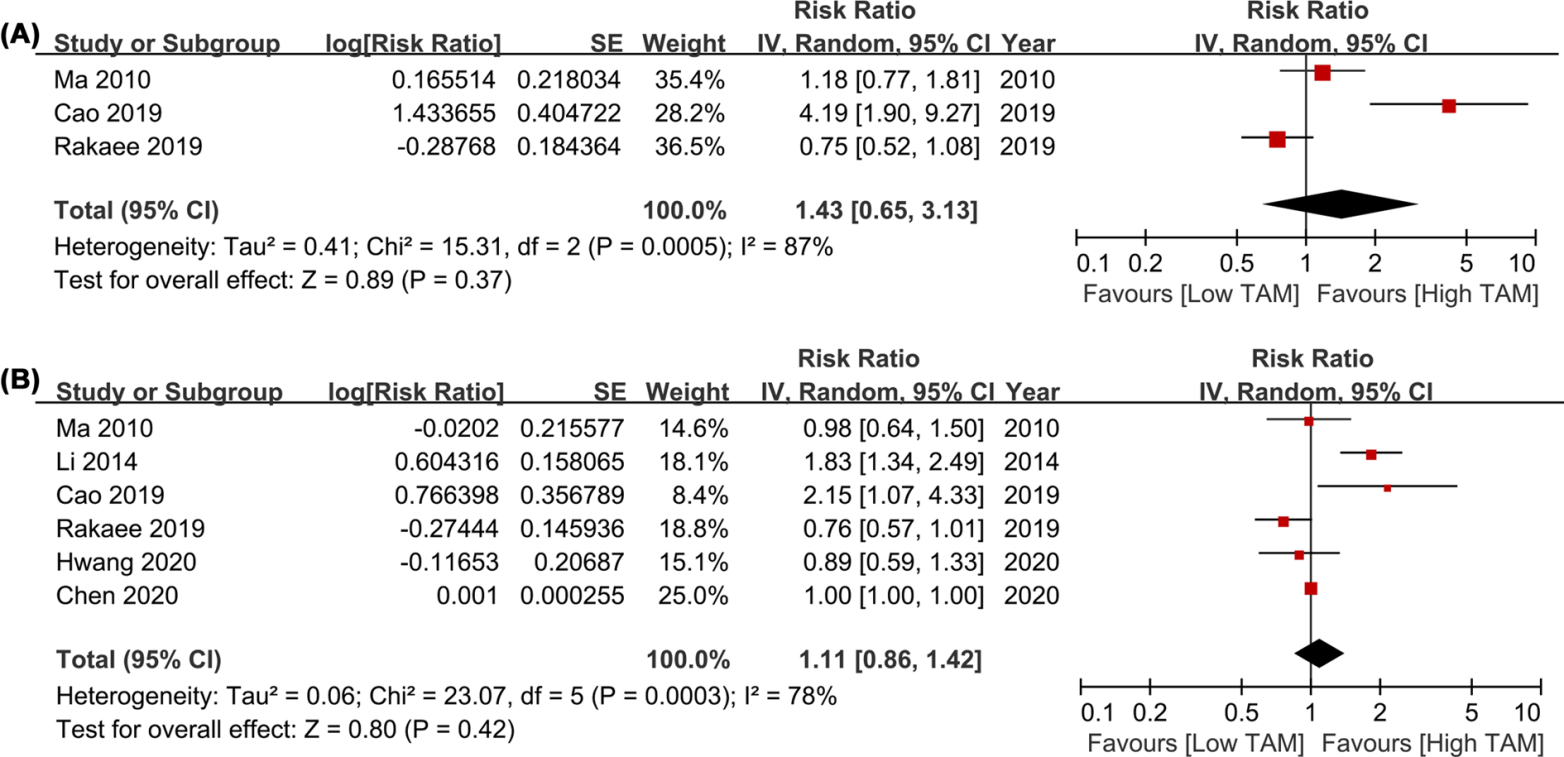
Forest plots comparing the survival of CD163+ TAMs in TN and TS for LCa patients (**A**) HR of OS for CD163+ TAMs in TN; (**B**) HR of OS for CD163+ TAMs in TS. Abbreviations: HR, hazard risk; LCa, lung cancer; OS, overall survival; TAMs, tumor-associated macrophages; TN, tumor nest; TS, tumor stroma.

This pooled analysis was performed in random-effect model for the significant heterogeneity in the followed results (all *I^2^ *≥ 50%). Three studies were included in the analysis of CD163+ TAMs in TN, and six studies were included to analyze the effect of CD163+ TAMs in TS on survival. Similar to CD163+ TAMs in TN (HR = 1.43, 95% CI = 0.65–3.13, *P=*0.37; *I^2^* = 87%; Figure 6A), the pooled HR of these studies showed that CD163+ TAMs infiltration was not associated with OS in TS (HR = 1.11, 95% CI = 0.86–1.42, *P=*0.42; *I^2^* = 78%; Figure 6B).

### Sensitivity analysis and publication bias

Due to the significant heterogeneity, we performed sensitivity analysis. Our analyses were robust in terms of the selection of the models and statistical methods. When the random-effect model was transformed into the fixed-effect model, the result showed that high CD68+ TAMs density in TS still predicted poor OS (HR = 1.31, 95% CI = 1.16–1.48, *P*<0.0001; *I^2^* = 71%). According to the funnel plot of the standard error by log RR, there was no significant publication bias in our study (Supplementary Figure S1).

## Discussion

Despite great developments in the rate of early diagnosis, the mortality of LCa has not been significantly improved, which calls for novel therapeutic modalities. TAMs, a hot topic in cancer researches, might become a promising target for LCa therapy [45]. Recent studies suggest that TAMs are closely linked to prognosis in patients with LCa [10,11,43]. However, few results have translated into clinical practice for the different conclusions among the previous studies. Hence, the present study seeks to assess the correlation of different markers and histologic locations for TAMs with LCa prognosis through pooling data from 30 eligible studies.

A total of 5105 patients were included in the present study. Our results suggested that the high CD68+ TAMs infiltration in TME was significantly associated with poor OS and DFS, whether identified in the tumor or TS. Likely, greater CD204+ TAMs density in TS suggested worse OS and DFS. On the contrary, high CD68+ TAMs density in TN predicted better OS, which was proved by the positive correlation between TN/TS ratio of CD68+ TAMs and OS. Besides, high HLA-DR+ TAMs density indicated better OS in TN and TS. However, neither nest CD163+ TAM density nor stromal CD163+ TAM density was correlated with OS in patients with LCa.

Traditionally, TAMs have been subdivided into two distinct macrophage phenotypes, proinfammatory M1 (classically activated macrophage) and anti-infammatory M2 (alternatively activated macrophage) [9]. M1 macrophages, which function as immune surveillance, exhibit antitumoral effects by serving as the antigen-presenting cell, secreting proinflammatory cytokines and chemokines, and largely express MHC class II (such as HLA-DR) [46]. This may explain that higher HLA-DR+ TAMs density indicated better OS in TI and TS in our study. In contrast, M2 macrophages, identified by the expression of CD204 (macrophage scavenger receptor class A) or CD163 (macrophage scavenger receptor class B), are considered to promote tumor progression by secreting multiple growth factors, proteolytic enzymes, and proangiogenic molecules [47,48]. It is the reason why greater CD204+ TAMs density in TS suggested worse OS and DFS. However, due to the limited studies included in our analysis, neither nest nor stromal CD163+ TAM density was correlated with OS.

CD68 is the most common biomarkers of TAMs. As for TAMs identification, 25 out of 30 included studies used CD68. Although CD68+ TAMs infiltration in the tumor was significantly associated with poor OS and DFS, the survival data of CD68+ TAMs in TS and TN were reversed. It is reported that the percentage of M1 TAMs was significantly lower than that of M2 TAMs in the LCa stroma [28]. Furthermore, a recent study revealed that more than half of TAMs in TN were M1 macrophages in the LCa [26]. The distinct distributions of M2 and M1 TAMs were in line with the different prognostic effects, namely, tumor promotors and tumor suppressors, which also could account for the positive correlation between TN/TS ratio of CD68+ TAMs and OS through pooled HR from univariate survival analysis and multivariate analysis.

In the present study, several important strengths should be acknowledged. We have extensively included studies about the TAMs on LCa prognosis to ensure that our results are more reliable. Moreover, the pooled analysis was performed to evaluate the effect of distinct TAMs markers (CD68, HLA-DR, CD163, and CD204) and histologic locations (TN +TS, TN, TS, and TN/TS) on the prognosis of LCa, including OS or DFS, as well as raw or adjusted measures, which made our study as comprehensive and persuasive as possible. Furthermore, the NOS scores of included studies were ranged from 6 to 9, and no publication bias has been detected, which ensured the validity of our results. Besides, our results found that TAMs were associated with LCa prognosis, which suggests that the TAMs may be a useful target for LCa.

The present study has the following limitations. For one thing, we strictly conducted the pooled analysis as detailed as possible to avoid confounding factors, including distinct TAMs markers, different histologic locations, OS or DFS, as well as raw or adjusted measures; therefore, the included studies were limited in some analyses. For another, some analyses could not be carried out further due to the limited data, such as pathological types of LCa, TAMs markers detection methods, therapies for LCa, the co-expression of markers for M1 or M2, the definition of TN and TS. Moreover, all included studies were retrospective study, which may lead to selection bias in the pooled results. In addition, the heterogeneity was significant in the present study, which required further studies with larger sample size to confirm the findings.

## Conclusion

In summary, this pooled analysis demonstrated the prognostic effect of TAMs on LCa patients. the high CD68+ TAMs infiltration in the tumor or TS indicated poor OS and DFS, while the higher CD68+ TAMs in TN or TN/TS ratio of CD68+ TAMs was associated with better prognosis. Besides, LCa patients with a high HLA-DR+ and low CD204+ TAMs density both had a long survival. Additional large-scale randomized controlled trials are remain needed to further investigate the clinical benefit.

## Supplementary Material

Supplementary Figure S1

## Data Availability

The datasets generated during and/or analyzed during the current study are available from the corresponding author on reasonable request.

## Abbreviations

CI: confidence interval
DFS: disease-free survival
EGFR: epidermal growth-factor receptor
HLA-DR: human leukocyte antigen DR
HR: hazard ratio
IHC: immunohistochemistry
KM: Kaplan–Meier
LCa: lung cancer
MHC: major histocompatibility complex
NOS: Newcastle–Ottawa Scale
OS: overall survival
PRISMA: Preferred Reporting Items for Systematic Reviews and Meta‐Analyses
TAM: tumor-associated macrophage
TME: tumor microenvironment
TN: tumor nest
TNM: tumor-node-metastasis
TS: tumor stroma
