# Supplementary material for: Prognostic significance of tumor-associated macrophages polarization markers in lung cancer: a pooled analysis of 5105 patients
Source: Biosci Rep. 2023 Feb 2;43(2):BSR20221659. doi: 10.1042/BSR20221659 (PMC9902841; doi:10.1042/BSR20221659)
Supplement: Supplementary Figure S1 [file BSR-2022-1659_supp.pdf]

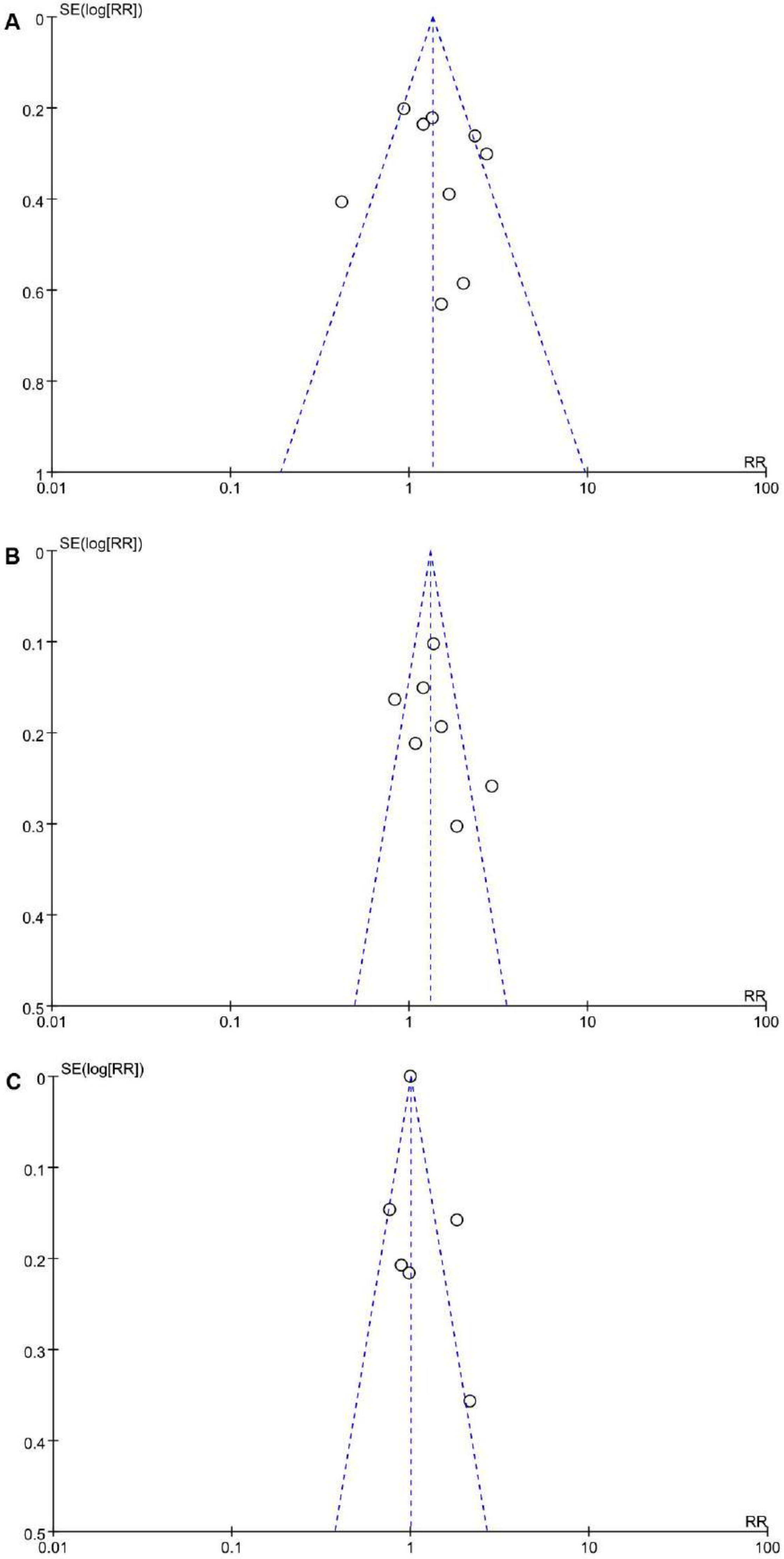
A
B
C
SE(log[RR])
RR
0
0.1
0.2
0.3
0.4
0.5
0.6
0.8
1
0.01
0.1
1
10
100

**Supplementary Figure S1** Funnel plot of studies with TAM density for potential publication bias assessment. (A) OS and CD68+ TAMs in the tumor; (B) OS and CD68+ TAMs in the tumor stroma; (C) OS and CD163+ TAMs in the tumor stroma.
